# A multilevel look at the construction of the monitoring and evaluation policy

**DOI:** 10.1590/0102-311XEN138426

**Published:** 2026-11-09

**Authors:** Ana Luiza d'Ávila Viana

**Affiliations:** 1 Faculdade de Medicina, Universidade de São Paulo, São Paulo, Brasil.

The article *Evaluation in Primary Health Care in Brazil: Reflections on Institutionalization* ^1^ by Eronildo Felisberto, Gabriella de Almeida Raschke Medeiros, Isabella Samico, Zulmira Maria de Araújo Hartz, Luciana Santos Dubeux, Ana Coelho de Albuquerque, and Juliana Martins Barbosa da Silva Costa introduces a current and strategic reflection by stating that the institutionalization of evaluation must go beyond the formal arrangements of health systems and seek deeper roots in the territories and in the relations between State and society toward a legitimized evaluation within the ethics of public service and social responsiveness.

This consideration alone - critically justified throughout the historical-institutional recovery of primary health care (PHC) evaluation and described in a synthetic, objective, clear and challenging framework - places this article as a watershed in the national literature on Monitoring and Evaluation (M&E).

Discussing the pitfalls in the policies to institutionalize evaluation, the authors (rigorously and with excellency and objectivity) list several challenges and point out where these dangers lie in the Brazilian national PHC evaluation policy, such as in the encapsulation of technical evaluation teams into isolated structures. They also draw attention to the need to train committed leaders, the concern with the continuity of resources and evaluative capacity, and the need to foster communities of learning and innovation practices, as well as ensuring expertise and providing opportunities for discussion forums. They conclude by describing possible dilemmas: the potentially contention-based character of evaluations in the face of political and/or managerial decisions; cultural changes promoted by stimuli toward dialogue and the unravel of controversies in dispute with the authoritarian character of management; and, finally, the need for funding evaluation processes in underfunded systems with recurrent spending cuts due to a long and permanent period of fiscal austerity. They also list three consequences that may result from institutionalization: the political aspect, the bureaucratic use, and the paradoxical character of evaluations.

The authors ^1^ also show that the scenario is confusing even in the face of clear regulations, taking as an example the ordinances that regulated the Brazilian National Primary Health Care Policy (PNAB, acronym in Portuguese) and defined the attributions of M&E actions. Although they recommend that state and federal managers should articulate strategies for institutionalizing evaluation, they neither establish consistent guidelines for structuring a policy nor provide for action plans, sources of financing, or sustainability mechanisms. Thus, the current use of evaluation as an instrument of control and its political use generate deleterious initiatives such as the demobilization of the Improvement Access and Quality National Program of Primary Health Care (PMAQ-AB, acronym in Portuguese) and the implementation of Previne Brasil. The authors’ assertive and courageous evidence shows how political conjunctures in scenarios of instability can generate false policies.

I want to draw attention to a point (later addressed by the authors) that I consider decisive for these incongruities: the lack of a multilevel view to evaluate a multilevel system, such as the Brazilian Unified National Health System (SUS, acronym in Portuguese). Put clearer: the absence of organizational and political legitimacy for a central component of complex systems, M&E. The institutionalization of the monitoring and evaluation policy ignores the federative issue (failing even to mention it). Such failure exacerbates several challenges; it could even solve many of them. PHC belongs to a multilevel system with intersections between scales and decision-making instances of the health system. Thus, the construction of the M&E policy must include discussion, negotiation, pacts, and agreements between the three levels of management and social control of the health system.

This federative look at the M&E policy remains central to its construction because what will be monitored and evaluated is a management shared by different levels of authority, forums, and instances of agreement, in addition to multiple arrangements, such as health consortia, fails to mention the institutions in the territories.

Thus, the M&E policy, rather than remaining exclusive to a level of management, must be shared by several actors in agreed and reviewed actions with guaranteed periodicity, creating permanent forums for discussion and decision.

Another important issue is the dissemination of results and the guarantee of their discussion at all levels and instances of the system, with the participation of universities in the territories and incorporated into new instances of macro-regional agreement, such as the Executive Committees for the Governance of Health Care Networks in the State of Bahia, for example.

As if these important contributions, reflections, and discussions of this article were insufficient, the authors also offer an analytical framework in which they clearly, objectively, and didactically introduce the paths of this innovative and transformative process of looking at M&E as an ethical issue in public service and an integral part of social responsiveness.

Based on an objective international literature review, the table by the authors points out important references for the construction of a conceptual framework for the M&E policy. I leave you with a suggestion: why not incorporate the issue of the resilience of national health systems and their multiple dimensions? After all, they undoubtedly constitute important aspects for a broader understanding of the territory, associated with the appreciation of ethical, professional, and social principles.

## Data Availability

Not applicable.
