# Supplementary material for: A multilevel look at the construction of the monitoring and evaluation policy
Source: Cad Saude Publica. 2026 Nov 9;42(Suppl 2):e00138426. doi: 10.1590/0102-311XEN138426 (PMC13750639; doi:10.1590/0102-311XEN138426)
Supplement: Translation

# Translation: Um olhar multinível para a construção da política de monitoramento e avaliação

**DOI:** 10.1590/0102-311XPT138426

**Authors:** Ana Luiza d'Ávila Viana

**Affiliations:** 1 Faculdade de Medicina, Universidade de São Paulo, São Paulo, Brasil.

O artigo *Avaliação na Atenção Primária à Saúde no Brasil: Reflexões sobre sua Institucionalização* ^1^, de Eronildo Felisberto, Gabriella de Almeida Raschke Medeiros, Isabella Samico, Zulmira Maria de Araújo Hartz, Luciana Santos Dubeux, Ana Coelho de Albuquerque e Juliana Martins Barbosa da Silva Costa, introduz uma reflexão atual e estratégica ao colocar que a institucionalização da avaliação deve ultrapassar os arranjos formais dos sistemas de saúde e buscar raízes nos territórios e nas relações entre Estado e sociedade - uma avaliação legitimada e que seja parte ética do serviço público e da responsividade social.

Somente essa consideração, justificada de forma crítica ao longo da recuperação histórico-institucional da avaliação da atenção primária saúde (APS) e apresentada em um quadro sintético, objetivo, claro e desafiador, já coloca esse texto como um divisor de águas na literatura nacional sobre Monitoramento e Avaliação (M&A).

Ao discorrer sobre as armadilhas presentes nas políticas de institucionalização da avaliação, os autores, de forma rigorosa e com grande qualidade e objetividade, enumeram diversos desafios e apontam, na política nacional de avaliação da APS, onde estão esses perigos, como no encapsulamento da equipe técnica da avaliação em estruturas isoladas. Além disso, chamam a atenção para a necessidade de formar lideranças comprometidas, a preocupação com a perenidade dos recursos e da capacidade avaliativa e a necessidade de fomentar comunidades de práticas de aprendizagem e inovação, bem como assegurar a presença de expertises e oportunizar fóruns de discussão. Concluem apresentando possíveis dilemas: o caráter potencialmente contestatório das avaliações frente a decisões políticas e/ou gerenciais; a mudança cultural promovida pelo estímulo ao diálogo e à exposição de controvérsias, em disputa com o caráter autoritário da gestão; e, finalmente, a necessidade de financiamento para os processos avaliativos em sistemas subfinanciados com recorrentes períodos de corte de gastos, dado um longo e permanente contexto de austeridade fiscal. Enumeram ainda três consequências que podem decorrer da institucionalização: o aspecto político, o uso burocrático e o caráter paradoxal das avaliações.

Evidenciam ainda os autores ^1^ que, mesmo diante de normativas claras, o cenário se mostra confuso, tendo como exemplo as portarias que regulamentaram a Política Nacional da Atenção Básica (PNAB) e definiram as atribuições das ações de M&A. Embora recomendem que estratégias de institucionalização da avaliação deverão ser articuladas pelos gestores estaduais e federal, não estabelecem diretrizes consistentes para a estruturação de uma política, tampouco preveem planos de ação, fontes de financiamento ou mecanismos de sustentabilidade. Constata-se, portanto, o uso corrente da avaliação como instrumento de controle e seu uso político, gerando iniciativas deletérias como a desmobilização do Programa de Melhoria do Acesso e da Qualidade da Atenção Básica (PMAQ-AB) e a implementação do Previne Brasil. Essa é uma evidência assertiva e corajosa dos autores, mostrando como as conjunturas políticas em cenários de instabilidade podem gerar falsas políticas.

Aqui quero chamar a atenção para um ponto, abordado mais à frente pelos autores, que considero decisivo também para essas incongruências: a falta de um olhar multinível para avaliar um sistema multinível, como o Sistema Único de Saúde (SUS). Mais claro: a ausência de legitimidade organizacional e política para um componente central dos sistemas complexos, o M&A. A questão federativa não é abordada na institucionalização da política de M&A - não é sequer citada -, e essa falha exacerba vários desafios e poderia, inclusive, ser a solução para muitos. Mesmo tendo como foco a APS, cabe salientar que ela está inserida em um sistema multinível, com pontos de interseção entre diferentes escalas e instâncias decisórias do sistema de saúde; portanto, a política de M&A não pode ser construída sem discussão, negociação, pactos e acordos entre os três níveis de gestão e controle social do sistema de saúde.

Esse olhar federativo para a política de M&A é central na construção da política, porque o que vai ser monitorado e avaliado é uma gestão compartilhada por diferentes níveis de autoridade, fóruns e instâncias de pactuação, além de múltiplos arranjos, como consórcios de saúde, sem contar as diferentes instituições presentes nos territórios.

A política de M&A, por isso, não pode ser exclusiva de um nível de gestão, mas sim compartilhada por diferentes atores em ações acordadas e revistas com periodicidade garantida, com a criação de fóruns permanentes de discussão e decisão.

Outra questão importante é a difusão dos resultados e a garantia de sua discussão em todos os níveis e instâncias do sistema, com a participação das universidades presentes em diferentes territórios e incorporadas em novas instâncias de pactuação macrorregionais, como os Comitês Executivos de Governança das Redes de Atenção à Saúde no Estado da Bahia, por exemplo.

Não bastassem essas importantes contribuições, reflexões e discussões do artigo, os autores propõem mais ao apresentar um quadro analítico no qual introduzem, de forma clara, objetiva e didática, os caminhos desse processo inovador e transformador de olhar para o M&A como uma questão ética no serviço público e parte integrante da responsividade social.

Com base em uma objetiva revisão bibliográfica internacional, o quadro elaborado pelos autores aponta referências importantes para a construção do marco conceitual da política de M&A. Deixo uma sugestão: por que não incorporar a questão da resiliência dos sistemas nacionais de saúde e suas múltiplas dimensões? Afinal, são, sem dúvida, importantes aspectos para a compreensão ampliada do território, associada à valoração de princípios éticos, profissionais e sociais.

## Data Availability

Não se aplica.
